# Bridging the gap: Gardner-Wells tongs utilization in pediatric spinal tuberculosis: A case report

**DOI:** 10.1016/j.ijscr.2024.110638

**Published:** 2024-11-22

**Authors:** Andra Hendriarto, Refky Juliandri, Bernadus Riyan Hartanto

**Affiliations:** Department of Orthopaedic and Traumatology, Dr. Cipto Mangunkusumo Hospital, Faculty of Medicine Universitas Indonesia, Indonesia

**Keywords:** Tuberculous spondylitis, Pott's disease, Gardner well tongs, Sternal occipital mandibular immobilizer

## Abstract

**Introduction:**

Spinal Tuberculosis (TB), or Pott's disease, is a significant form of extrapulmonary TB affecting the spine, especially in children. Standard treatments include anti-tuberculosis medications, immobilization, and surgery. The use of Gardner well tongs (GWT) in pediatrics spinal TB is rare due to associated risks and lack of supporting evidence. This case report aims to document the application of GWT traction in conjunction with surgical intervention for a pediatric patient with tuberculosis spondylitis.

**Case presentation:**

An 8-year-old boy presented with progressive weakness in both legs over 2.5 years, culminating in his inability to stand or walk. Physical examination revealed asymmetrical back, gibbus formation, tenderness, limited range of motion, and upper motor neuron neurological deficits. Radiographic imaging showed vertebral body destruction from T1–6, causing an 81-degree kyphotic curve. The patient underwent 5 kg of GWT traction for 2 weeks, resulting in a 10-degree improvement in curvature. Subsequent surgical procedures included laminectomy, posterior stabilization, deformity correction, and post-operative application of a SOMI brace. By discharge, the patient's kyphotic angle had improved from 81 to 63°, and there was notable improvement in motor strength and neurological function.

**Discussion:**

While surgical intervention is often necessary for vertebral deformity restoration, GWT offers advantages in spinal TB management, such as achieving stable cervical segments without skin incision and aiding gradual kyphotic correction. Serious complications from GWT, like skull perforation or neurovascular damage, are infrequent.

**Conclusion:**

A comprehensive, holistic approach incorporating GWT traction and surgical intervention is essential for improving clinical outcomes in pediatric tuberculosis spondylitis.

## Highlights

• Spinal Tuberculosis, is a form of extrapulmonary TB that affects the spine, particularly in pediatric populations.
• Spinal TB treatment in pediatric patients focuses on anti-tuberculosis medication, immobilization, and surgical intervention.
• The use of Gardner well tongs (GWT) is not commonly chosen for treatment due to its risk and lack of evidence.
• Up to day, there is still no case report that reported the usage of GWT in pediatric patient with spinal tuberculosis.
• This report aims to share our experience in using GWT traction with surgical intervention for managing pediatric spinal TB.

## 1 Introduction

Paraplegia and kyphotic deformity due to tuberculosis spondylitis (TS) are serious complications that can lead to significant morbidity and disability. Tuberculosis spondylitis, also known as Pott's disease, is a form of osteoarticular tuberculosis that can result in the destruction of the vertebral body, leading to kyphotic deformity and neurological deficits such as paraplegia. It accounts for approximately 50 % of all skeletal TB cases, especially in children. All regions of the spine can be affected by TS, however, the most commonly affected area is the thoracolumbar junction [1,2]. While typically the disease is treatable with a combination of antituberculous drug regimen, in some cases, the use of Gardner-Wells tongs (GWT) and surgical intervention may be necessary to treat these complications [3,4].

Gardner-Wells tongs, also known as halo vests, are a type of external fixation device used to immobilize the spine and provide stability to the affected area. This device can be used in the treatment of spinal tuberculosis, as it allows for the application of external forces to correct the kyphotic deformity and provide stability to the spine. Surgical intervention may also be required in cases of severe spinal tuberculosis, such as when there is spinal cord compression, the presence of neurological deficits, or when the kyphotic deformity is significant. Various surgical techniques, including ventral, combined, and dorsal approaches, can be used to correct the kyphotic deformity and provide stability to the spine [[3], [4], [5]].

In this paper, we reported a case of an 8-year-old boy with complaints of progressive weakness on both legs since 2,5 years ago. The weakness was also accompanied by growing kyphotic deformity on the child's thoracal spine region. In an attempt to improve his condition, we decided to perform cervical traction using GWT system for two weeks followed by total treatment VI which consisted of laminectomy, posterior stabilization, deformity correction, and also deep speciment culture and biopsy. We applied Sternal Occipital Mandibular Immobilizer (SOMI) brace post-operatively. This paper highlights the importance of considering surgical intervention in such cases, as well as to depict the use of GWT system to reduce the degree of kyphotic deformity and the benefits of using SOMI brace in postoperative spine cases.

## 2 Case presentation

A 8 year-old boy came to the hospital complaining of weakness on both legs since 2.5 years ago. In 2021, patient complained for weakness on both legs since 2.5 years. Around a few days after the onset of weakness, there was a complaint of back pain. The weakness became progressively worse within a month, and eventually he can't to stand and walk. In September 2023, the patient underwent a spinal MRI, with the results suggesting a spinal infection. The patient was then referred to RSCM for further management. Surgery on the spine was recommended. Patient denied any history of chronic cough and fever but he has history of weight loss 4.5 kg in 3 months. His micturition and defecation are within normal limit and the patient had never received anti-tuberculosis medication.

**Fig. 1:**
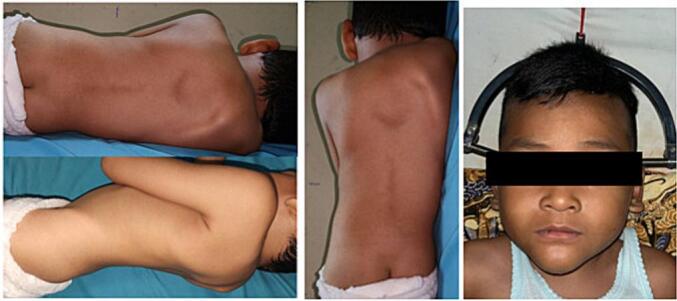
Local Status of the patient's back, showing severe kyphotic of 81°.

Physical examination showed asymmetrical back with gibbus appearance without any apparent wound (Fig. 1), patient also felt tenderness upon palpation with VAS 1–2 on thoracal region. When instructed to move his back, the patient has limited range of motion from the pain elicited with movement. Neurologic examination showed increasing reflex on the patellar and Achilles tendon on both sides, positive Babinski reflex, positive clonus, and negative Lhermitte, Hoffman and Trommer sign. Patient felt pain on right T3, bilateral T4 and left side of T5.

**Fig. 2:**
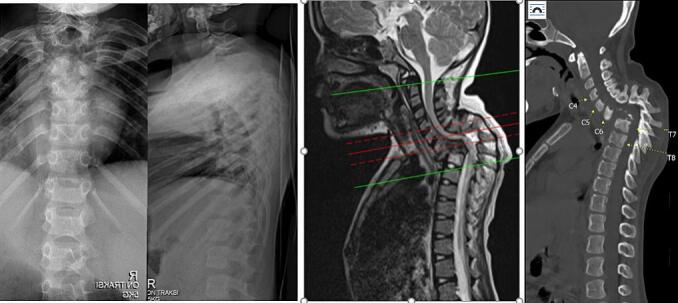
Radiologic examination on the patient, (A) thoracolumbal view of plain radiographic, (b) Sagittal view of non-contrast cervicothoracal MRI, (c) Sagittal view of cervicothoracal CT Scan.

Radiographic result of thoracal X-ray showed kyphotic deformity around 81° with no listhesis, destructed bone from Th1-Th6, end plate cartilage destruction, and no joint space narrowing, to which conclude that the destructed Th1-Th6 manifested in kyphotic deformity in the patient. Cervical imaging using X-ray showed tuberculous spondylitis at C4-C7 level with kyphotic deformity on cervicothoracal vertebrae. MRI with contrast on the whole spine highlighted kyphotic curve with peak at C7-Th1, compression and edema at C7, Th1 with soft tissue edema, moderate canal stenosis due to suspected tuberculous spondylitis. From these workups, patient was diagnosed with paraplegia due to tuberculous spondylitis of C7-Th2 Frankel B with paravertebral abscess and kyphotic deformity (Fig. 2).

**Fig. 3:**
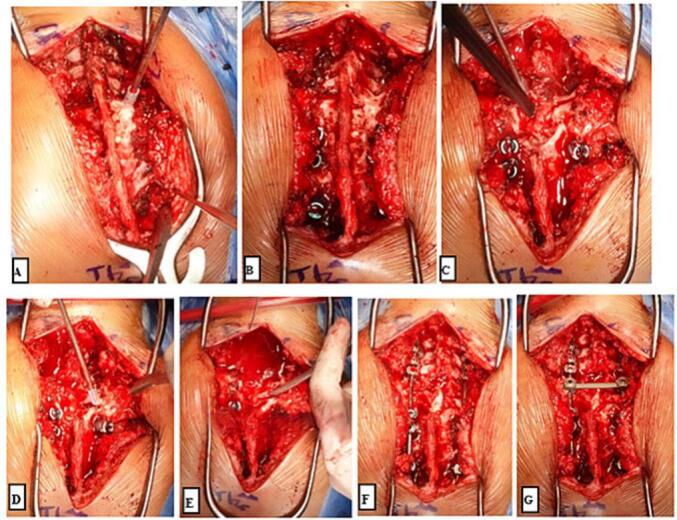
Intraoperative image of Total Treatment VI management. (a) Exposure of Cervicothoracal, (b) pedicle screw insertion, (c) Laminectomy of C7-T5, (d) Ponte Osteotomy, (e) Deep specimen and biopsy, (f) Rod insertion and cantilever technique, (g) Final construct.

**Fig. 4:**
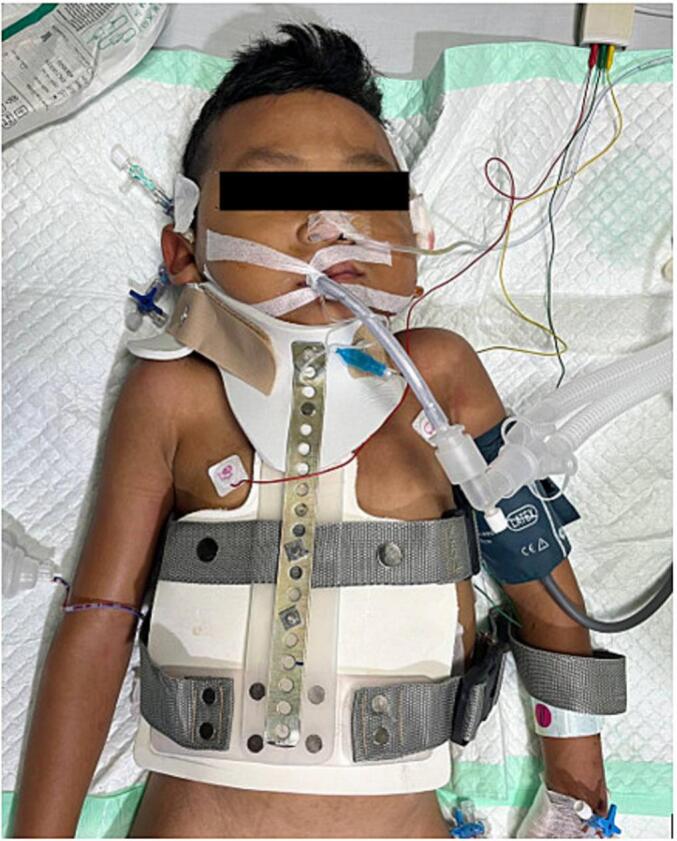
Postoperative use of SOMI Brace application.

**Fig. 5:**
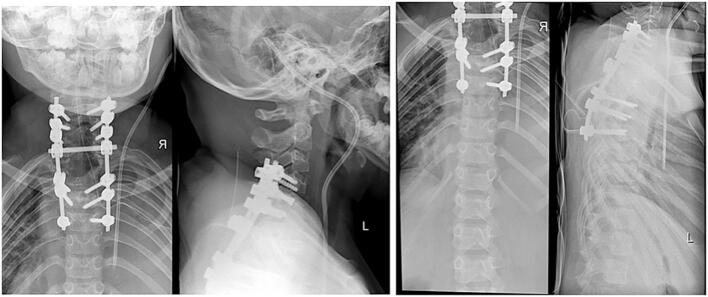
Postoperative X-ray of the cervical and thoracal, AP and lateral.

**Fig. 6:**
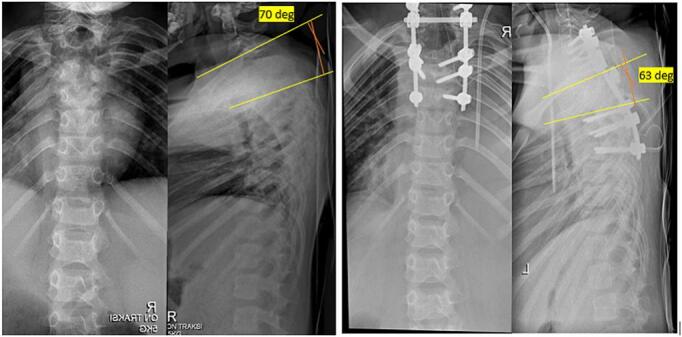
Preoperative and postoperative comparison of kyphotic angle, whereby preoperative kyphotic angle was 70° and postoperative kyphotic angle reached 63°.

As the initial management, patient was put on Gardner-Wells tongs (GWT) for 2 weeks prior to receiving surgery, which gave improvements to patient's kyphotic angle by 11^o^. Afterwards, patient underwent Total Treatment VI series of treatment, which consist of Debridement, Abscess evacuation, Deep specimen culture and biopsy, Open decompression of C7-T2 and Posterior stabilization C5-C6, T3-T4, deformity correction (Fig. 3). Following the surgery, the physician applied Sternal Occipital Mandibular Immobilizer (SOMI) brace (Fig. 4). The postoperative radiography revealed posterior stabilization devices spanning from C5-C6, and the kyphotic angle decreased by 7^o^ from 70^o^ preoperatively to 63^o^ (Figs. 5, 6). The case report was made in line with the SCARE criteria [6].

## 3 Discussion

Cervical tuberculous spondylitis poses a significant risk of spinal cord compression, which can lead to severe neurological deficits, including quadriplegia. Early diagnosis is crucial for effective management, as delays can result in irreversible damage. Surgical intervention is often warranted in cases where conservative treatment fails or when there is evidence of spinal instability or neurological deficits. The Gardner-Wells tongs, a method of cervical traction, are frequently employed to stabilize the cervical spine and alleviate pressure on the spinal cord during the surgical procedure. This technique has been shown to be effective in providing temporary stabilization, particularly in cases of cervical dislocation or fracture [[7], [8], [9]].

This patient came with neglected case of tuberculous spondylitis and presented with severe neurological deficit along with kyphotic back. As the kyphotic angle is considerably severe (>60°) and resulted from tuberculous infection, kyphosis improvement became the focus of treatment in the active stage. In addition to neurological deficit, abscess on the paravertebral region, instability of the spine from kyphotic deformity and anti-tuberculous chemotherapy resistances are some of the indications for surgical management for tuberculous spondylitis [10,11].

Gardner Wells tongs are a temporary traction device commonly used in cervical spine injuries and surgeries. They consist of tongs with skull pins that are used to pull the head away from the torso, applying traction force along the axis of the cervical spine [8]. These tongs are utilized for stabilizing the head and maintaining or improving cervical sagittal alignment, making them valuable in cases requiring cervical traction [12]. The use of Gardner Wells tongs, along with other traction methods like bivector traction, has been reported to be safe and effective in stabilizing the head and achieving optimal cervical alignment in various spinal procedures, as it provides not only stabilization, but also deformity correction and head elevation, which eventually leads to enhanced surgical correction of spinal malformations [13,14].

The experience with Gardner Wells tongs in tuberculous spondylitis cases involves their application for stabilizing the head and maintaining proper cervical alignment during surgical interventions. These tongs play a crucial role in providing traction and stability, especially in cases where maintaining cervical alignment is essential for successful outcomes. The use of Gardner Wells tongs, along with advancements in spinal instrumentation, has contributed to improved safety and efficacy in the treatment of tuberculous spondylitis, particularly in achieving adequate stabilization and alignment of the cervical spine during surgical procedures. The use of Gardner Well tongs for tuberculous spondylitis has been reported in one case of adult patient with cervical tuberculous spondylitis and severe neurologic deficit of spinal quadriplegia. Although it was not mentioned regarding the duration of GWT use, the patient underwent three stage of surgery and eventually were able to gain neurologic function restoration and able to return to exercise. [9,15]

Gardner-Wells tongs can be effectively utilized for both preoperative stabilization and intraoperative traction in children with cervical spine pathologies, including tuberculous spondylitis. For instance, a case study highlighted the successful use of Gardner-Wells tongs to achieve closed reduction of a dislocated cervical facet joint in a 9-year-old boy, demonstrating the efficacy of this method in pediatric cases. Furthermore, the systematic review by Saleh et al. emphasizes that when applied correctly, Gardner-Wells tongs can be a safe and effective method for cervical traction, even in pediatric populations. However, the use of Gardner-Wells tongs in younger patients, particularly those under the age of 1 to 2 years, is generally limited due to concerns regarding the unfused cranial sutures and the thinness of the calvarium, which increases the risk of complications [9,16,17].

Despite its vital role in managing cervical defects, there are complications of GWT use that may emerge. The complication rate of GWT use ranges from 12.5 to 37.5 %, and this number came from a small-sized studies published on the use of GWT (*n* = 16), from which the complications including loosening pins, asymmetric pins and infection [9,18].

The duration for neurological improvement after surgical treatment of tuberculous spondylitis varies depending on the specific case and the extent of neurological deficits. Studies have reported different timelines for neurological improvement following surgical intervention for spinal tuberculosis. In a study by Isaack [19], patients showed an improvement in kyphosis of 13° and neurological improvement by one Frankel grade at a 30-month follow-up. This indicates that neurological improvement can be observed over an extended period after surgery for tuberculous spondylitis. Additionally, Lee et al [ 20] reported that for pyogenic spondylitis, clinical signs, including neurological manifestations, typically appeared after an average of 6.4 months. This timeline may offer some insight into the potential duration for neurological improvement in cases of tuberculous spondylitis as well. Moreover, the study by assessed patients at 3, 6, and 12 months post-surgery and then annually thereafter, highlighting the importance of monitoring neurological improvement and other outcomes in the post-operative period [21]. This longitudinal follow-up suggests that regular assessments are crucial to track progress and ensure optimal outcomes in patients undergoing surgical intervention for spinal tuberculosis.

Various surgical methods are employed to manage spinal tuberculosis, each with specific advantages and disadvantages. Anterior decompression and fusion involves removing infected tissue and stabilizing the spine, achieving success rates of 80–90 %, especially effective in localized infections. Posterior decompression and instrumentation, with success rates of 75–85 %, is beneficial when the anterior column is compromised. A combined anterior-posterior approach offers comprehensive treatment, yielding success rates of 85–95 %, particularly in severe cases. Debridement with internal fixation using titanium mesh provides 80–90 % success in stabilizing the spine. Percutaneous techniques are less invasive but lack detailed pediatric success rates [[22], [23], [24]].

In addition to GWT, this case use SOMI brace as postoperative fixation. The SOMI brace is a type of cervicothoracic brace used for immobilization and stabilization of the cervical spine. It consists of a rigid structure that extends from the sternum to the occiput and mandible, providing support and restricting motion in the cervical region [25]. The SOMI brace is indicated for various conditions requiring cervical immobilization, such as cervical spine injuries, fractures, instability, or postoperative management [[26], [27], [28]]. It is particularly useful in cases of odontoid fractures, where immobilization is essential for proper healing and alignment [28]. With the help of both orthopaedics apparatuses, we expected the patient to obtain proper management of neglected cervical deformities as the result of tuberculous spondylitis.

## 4 Conclusion

In addition to anti-tuberculous chemotherapy and surgery, the role of external aids and hardware such as GWT and SOMI brace have shown their contribution in further improving the degree of kyphotic deformity and restore motoric strength in the lower extremities. The case highlights the importance of comprehensive and holistic approaches in managing tuberculosis spondylitis, including the use of external fixation devices and surgical intervention.

## Ethical approval

Ethical approval was not required in the treatment of the patient in this report as the patient's personal identity were concealed, hence it was exempted from obtaining ethical approval from the ethics committee.

## Sources of funding

The authors report no external source of funding during the writing of this article.

## Author's contribution

Conceptualization: [Andra Hendriarto, Refky Juliandri, Indra Kusuma Jaya, Bernadus Riyan Hartanto]; Methodology: [Andra Hendriarto]; Project Administration: [Refky Juliandri]; Formal analysis and investigation: [Bernadus Riyan Hartanto]; Resources:[Indra Kusuma Jaya]; Visualization: [Indra Kusuma Jaya]; Writing-original draft preparation:[Refky Juliandri]; Writing-review and editing: [Bernadus Riyan Hartanto, Andra Hendriarto]; Supervision:[Andra Hendriarto].

## Guarantor

Refky Juliandri: accepts full responsibility for the work and/or the conduct of the study, has access to the data, and controlled the decision to publish.

## Research registration

(for case reports detailing a new surgical technique or new equipment/technology)

NA.

## Consent

Written informed consent was obtained from the patient's parents/legal guardian for publication and any accompanying images. A copy of the written consent is available for review by the Editor-in-Chief of this journal on request. Patients' name and personal identity remained anonymous and omitted if it is not necessary.

## Provenance and peer review

Not commissioned, externally peer-reviewed.

## Summary statement

No funding was received to assist with the preparation of this manuscript. All the data are available can be accessed via corresponding email after clearly stating the intention and permission to conduct research that requires our data. All authors in this research have given consent to participate in this manuscript and agree to this current form of manuscript to be submitted for publication.

## Declaration of competing interest

The authors declare that there is no conflict of interest regarding the publication of this paper.

## References

[1] Garg R.K., Somvanshi D.S. (2011). Spinal tuberculosis: a review. J. Spinal Cord Med..

[2] Leowattana W., Leowattana P., Leowattana T. (2023).

[3] Soeroso N.N., Pradana A., Lubis N., Soeroso L. (2018 Aug 1). Successful treatment of total paraplegic patient due to tuberculous spondylitis. Respirol Case Rep..

[4] Shengfa P., Hongyu C., Yu S., Fengshan Z., Li Z., Xin C. (2023 Jan). Effect of cervical suspensory traction in the treatment of severe cervical kyphotic deformity. Front. Surg..

[5] Saleh H., Yohe N., Razi A., Saleh A. (2018 Mar). Efficacy and complications of the use of Gardner-Wells tongs: a systematic review. Journal of Spine Surgery..

[6] Sohrabi C., Mathew G., Maria N., Kerwan A., Franchi T., Agha R.A. (2023 May 1). The SCARE 2023 guideline: updating consensus Surgical CAse REport (SCARE) guidelines. Int J Surg [Internet].

[7] Prihartomo G.A. (2023). Cervical tuberculous spondylitis. Asian Australasian Neuro and Health Science Journal (Aanhs-J)..

[8] Hammond J.M., Tarakji B.A., Frank C.A., Stewart T.F., Fernandez D., Atkinson P. (2019). Traction load, tong position, and head support significantly influence cervical spine loading during traction. Proc. Inst. Mech. Eng. H.

[9] Saleh H, Yohe N, Razi A, Saleh A. Efficacy and complications of the use of Gardner-Wells Tongs: a systematic review. Journal of Spine Surgery [Internet]. 2018 Mar [cited 2024 Apr 9];4(1):123–9. Available from: https://jss.amegroups.org/article/view/4063/html. 10.21037/jss.2018.03.03 PMC5911751 29732432

[10] Rasouli MR, Mirkoohi M, Vaccaro AR, Yarandi KK, Rahimi-Movaghar V. Spinal tuberculosis: diagnosis and management. Asian Spine J [Internet]. 2012 Dec 31 [cited 2024 Apr 9];6(4):294–308. Available from: https://pubmed.ncbi.nlm.nih.gov/23275816/. 10.4184/asj.2012.6.4.294 PMC3530707 23275816

[11] Soeroso N.N., Pradana A., Lubis N.D., Soeroso L. (2018). Successful treatment of total paraplegic patient due to tuberculous spondylitis. Respirol. Case Rep..

[12] Abolfotouh S.M., Moore D.K. (2019). Use of simultaneous traction over a halo ring to achieve reduction of a type 2 odontoid fracture for anterior odontoid screw fixation. Int. J. Surg. Case Rep..

[13] Karikari I.O., Bumpass D.B., Gum J.L., Sugrue P.A., Chapman T.M., Elsamadicy A.A. (2017). Use of bivector traction for stabilization of the head and maintenance of optimal cervical alignment in posterior cervical fusions. Global Spine J..

[14] Koreckij J., Price N., Schwend R.M. (2011 Jul 1). Vectored cranial-cervical traction limits facial contact pressure from prone positioning during posterior spinal deformity surgery. Spine (Phila Pa 1976) [Internet].

[15] Adeleke AA, Komolafe EO, Dada OA, Owagbemi OF. Spinal decompression with 360° instrumented fusion for unstable tuberculous quadriplegia in a young adult—a case report. J Biosci Med (Irvine) [Internet]. 2015 [cited 2024 Apr 9];03(08):37–43. Available from: http://file.scirp.org/Html/.

[16] Parada S.A., Arrington E.D., Kowalski K.L., Molinari R. (2010). Unilateral cervical facet dislocation in a 9-year-old boy. Orthopedics.

[17] Menger R., Beauchamp E.C., Alexiades N.G., Szpilka R.T., Anderson R.C.E. (2023). Neonatal halter traction for severe cervical spine deformity: a technical case report with 2-year follow-up. Operative Neurosurgery.

[18] Kepler CK, Vaccaro AR, Chen E, Patel AA, Ahn H, Nassr A, et al. Treatment of isolated cervical facet fractures: a systematic review. J Neurosurg Spine [Internet]. 2016 Feb 1 [cited 2024 Apr 9];24(2):347–54. Available from: https://pubmed.ncbi.nlm.nih.gov/26516667/. 10.3171/2015.6.SPINE141260 26516667

[19] Issack P.S., Boachie-Adjei O. (2011). Surgical correction of kyphotic deformity in spinal tuberculosis. Int. Orthop..

[20] Lee K.Y. (2014). Comparison of pyogenic spondylitis and tuberculous spondylitis. Asian Spine J..

[21] Debnath U.K., McConnell J.R., Kumar S.V.A. (2021). Single-stage combined anterior corpectomy and posterior instrumented fusion in tuberculous spondylitis with varying degrees of neurological deficit. Int. J. Spine Surg..

[22] Liu J., Wan L., Long X.H., Huang S.H., Dai M., Liu Z. (2015). Efficacy and safety of posterior versus combined posterior and anterior approach for the treatment of spinal tuberculosis: a meta-analysis. World Neurosurg..

[23] Zhang H., Zeng K., Yin X., Huang J., Tang M., Guo C. (2015). Debridement, internal fixation, and reconstruction using titanium mesh for the surgical treatment of thoracic and lumbar spinal tuberculosis via a posterior-only approach: a 4-year follow-up of 28 patients. J Orthop Surg Res..

[24] Buyukbebeci O., Şeçkiner İ., Karslı B., Karakurum G., Baskonus H.M., Bilge O. (2011). Retroperitoneoscopic drainage of complicated psoas abscesses in patients with tuberculous lumbar spondylitis. Eur. Spine J..

[25] Dakhil-Jerew F., Derry F. (2008). Management of cervical spine fracture in patient with advanced ankylosing spondylitis using SOMI brace. Injury Extra.

[26] Ryken T.C., Hadley M.N., Aarabi B., Dhall S.S., Gelb D.E., Hurlbert R.J. (2013). Management of acute combination fractures of the atlas and axis in adults. Neurosurgery.

[27] Su Y., Wei B., Tian Y., Jiang Y., Xu W., Wang L. (2018). The comparison of clinical outcome of fresh type II odontoid fracture treatment between anterior cannulated screws fixation and posterior instrumentation of C1-2 without fusion: a retrospective cohort study. J. Orthop. Surg. Res..

[28] Su Y., Wei B., Tian Y., Jiang Y., Xu W., Wang L. (2018). Posterior temporary C1-2 fixation for 3-part fractures of the axis (odontoid dens and hangman fractures). Medicine.

